# An optical aptasensor for real-time quantification of endotoxin: From ensemble to single-molecule resolution

**DOI:** 10.1126/sciadv.adf5509

**Published:** 2023-02-08

**Authors:** Pancheng Zhu, Vasileios A. Papadimitriou, Jeanne E. van Dongen, Julia Cordeiro, Yannick Neeleman, Albert Santoso, Shuyi Chen, Jan C. T. Eijkel, Hanmin Peng, Loes I. Segerink, Alina Y. Rwei

**Affiliations:** ^1^ Department of Chemical Engineering, Delft University of Technology, 2629 HZ, Delft, Netherlands.; ^2^ State Key Laboratory of Mechanics and Control of Mechanical Structures, Nanjing University of Aeronautics and Astronautics, 210016 Nanjing, China.; ^3^ BIOS Lab on a Chip Group, MESA+ Institute for Nanotechnology, Technical Medical Centre, Max Planck Institute for Complex Fluid Dynamics, University of Twente, P.O. Box 217, 7500 AE Enschede, Netherlands.; ^4^ Institute of Organic and Polymeric Materials, National Taipei University of Technology, 10608 Taipei, Taiwan.; ^5^ Research and Development Center for Smart Textile Technology, National Taipei University of Technology, 10608 Taipei, Taiwan.

## Abstract

Endotoxin is a deadly pyrogen, rendering it crucial to monitor with high accuracy and efficiency. However, current endotoxin detection relies on multistep processes that are labor-intensive, time-consuming, and unsustainable. Here, we report an aptamer-based biosensor for the real-time optical detection of endotoxin. The endotoxin sensor exploits the distance-dependent scattering of gold nanoparticles (AuNPs) coupled to a gold nanofilm. This is enabled by the conformational changes of an endotoxin-specific aptamer upon target binding. The sensor can be used in an ensemble mode and single-particle mode under dark-field illumination. In the ensemble mode, the sensor is coupled with a microspectrometer and exhibits high specificity, reliability (i.e., linear concentration to signal profile in logarithmic scale), and reusability for repeated endotoxin measurements. Individual endotoxins can be detected by monitoring the color of single AuNPs via a color camera, achieving single-molecule resolution. This platform can potentially advance endotoxin detection to safeguard medical, food, and pharmaceutical products.

## Teaser

Researchers developed an aptamer-based endotoxin optical sensor for real-time endotoxin detection with single-molecule resolution.

## INTRODUCTION

Endotoxin is a lipopolysaccharide (LPS) constituting the outer membrane of Gram-negative bacteria (*1*). It is a pyrogen that is extremely toxic to humans. This is evidenced by the fact that endotoxin is a main trigger of sepsis, a disease that accounts for one of five deaths in intensive care units and a mortality rate of 42% (*2*, *3*). Strict monitoring of endotoxin levels is therefore crucial and mandatory in industrial practices (e.g., food and pharmaceutical industries).

Now, the most sensitive and specific method for endotoxin detection is the limulus amebocyte lysate (LAL) assay, a batch-based process with limitations including (i) the inability to be used for repeated detection, (ii) unsustainability due to the extensive use of blood from horseshoe crabs (*4*), (iii) long preparation times, and (iv) high susceptibility to human errors (*5*). In view of the aforementioned drawbacks of the LAL assay and the high demand from food and pharmaceutical industries, substantial efforts are being made to design alternative endotoxin sensors that are direct, specific, and able to detect LPS repeatedly. Among the bioreceptors used in sensors to detect specific biotargets, antibodies are the gold standard (*6*). Nevertheless, antibodies are inherently unstable and have a costly and cumbersome synthesis process (*7*). Therefore, a sensing technique that is direct, versatile, and able to detect endotoxin repeatedly in a short time frame will be of high significance.

Aptamer biosensors (i.e., aptasensors) have attracted substantial attention for the detection of a wide variety of biological molecules ranging from DNA and proteins to metabolites and small molecules (*8*). Aptamers are single-stranded DNA (ssDNA) or RNA molecules with three-dimensional conformational structures interacting with specific biomolecules of interest (*8*, *9*). Aptamers can be designed for a wide variety of target molecules by changing the nucleic acid sequence (*10*–*13*). Compared to traditional ligands such as antibodies, aptamers have higher specificity, better stability, high affinity, and are easier to manufacture and, therefore, less expensive (*9*).

The readout methods for aptasensors can be roughly divided into two categories: optical (*14*) and electrochemical detection (*15*). Optical-based aptamer sensors have the advantage of higher durability, extended stability, absence of electromagnetic interference, and smaller dimensions when compared with their electrochemical counterpart (*16*). Single-molecule sensing is defined by the ability of a sensor to generate one signal for one molecule; this is in contrast with ensemble sensing, in which one signal is generated by a collection of molecules (*17*). Single-molecule sensing has advantages in accuracy, high signal-to-noise ratio, and ability to take into account the variability of individual molecules (*17*–*19*); a technology suitable for the optical detection of endotoxin with single-molecule resolution would be of scientific interest. This presents an avenue to further understand the individual variability of the detected analytes, generating insights that would otherwise be absent in ensemble averaging mode. Nevertheless, current technologies in endotoxin detection are limited to ensemble resolution (*20*–*21*). A sensor with single-molecule resolution would further enhance the development of this field.

Localized surface plasmon resonance (LSPR)–based sensors could be the tool for realizing endotoxin sensing with single-molecule resolution: LSPR-based sensors are recognized for their high resolution while offering relatively simple readout. In addition, the technology is label free and captures the real-time interaction of the target molecule with the bioreceptor (*22*). Plasmonic materials also have the advantage of a high scattering cross section and a high photostability due to their insusceptibility toward photobleaching. The aim of this work is to develop an LSPR-based sensor suitable for the repeated detection of endotoxin with single-molecule resolution.

**Fig. 1.**
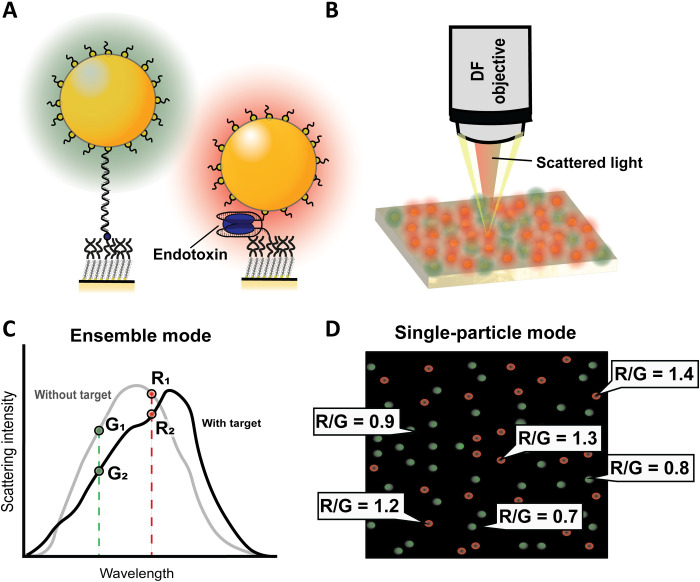
Schematic overview of the sensing process. (**A**) An ssDNA aptamer molecule is bound to the AuNF and AuNP. Upon target binding, the aptamer molecule will interact with endotoxin, upon which the average z-height of the AuNP decreases and induces a redshift of the scattering wavelength. (**B**) Measurement principle of the AuNPs on the AuNF, where light is emitted on the film and scattered light is captured by a dark-field (DF) microscopy objective. (**C**) Determination of the red/green (RG) ratio from the averaged-spectral information in the ensemble mode. (**D**) Single-particle mode, where the RG ratio is determined for all individual particles, which allows for sensing with individual endotoxin-binding resolution.

This paper reports an aptamer-based sensing technology that relies on the z-height–dependent electromagnetic coupling of gold nanoparticles (AuNPs) to a gold nanofilm (AuNF) (Fig. 1). The sensor consists of AuNPs tethered to a AuNF by a single aptamer that can specifically bind to endotoxin (Fig. 1). By using a subcategory of aptamers that undergoes conformational changes upon binding with the target analyte *(**23*–*27*), the z-height of the AuNP can be related to the binding of endotoxin. The aptamer acts as a molecular ruler that controls the nanoscale distance between the AuNP and the AuNF: In its unbound state, the aptamer is a flexible ssDNA tether; however, upon endotoxin binding, part of the ssDNA sequence folds around the endotoxin. This decreases the time-averaged distance between the AuNP and the AuNF (*28*). Since the plasmon resonance coupling is distance dependent, endotoxin binding results in the red shifting of the scattered light spectrum (see the Supplementary Materials for details) (Fig. 1A).

In ensemble measurements, as shown in Fig. 1C, the sum of the scattering spectra of all the AuNPs is used to determine the red/green ratio (RG ratio). In the ensemble mode, the presented sensor exhibits high selectivity, high reliability, and can be regenerated for repeated usage, which may be further developed for continuous detection. In the single-particle mode, the RG ratio of individual AuNPs is assessed, as shown in Fig. 1D, which is successfully demonstrated in this work. In this platform, the response of one AuNP indicates the response toward one endotoxin molecule; therefore, single-molecule resolution can be achieved via the single-particle mode. In summary, this study features an optical aptasensor that can be used in two different measurement modes for selective and repeatable endotoxin detection.

## RESULTS

### Sensor preparation and verification via XPS, QCM, and SEM

Figure S1 illustrates the sensor fabrication process (see details in the Supplementary Materials). AuNP density was controlled by the ─OH and ─COOH end groups of the self-assembled monolayer (SAM) (fig. S2). In principle, the more AuNPs tethered to the AuNF via the aptamer, the lower the concentration of endotoxin one could measure: If only a small fraction of aptamers will bind endotoxin in equilibrium, then the number of aptamer-tethered AuNPs on the sensor defines whether the number of binding events can be considered statistically relevant (*29*). This directly influences the sensor’s sensitivity (i.e., ability of the sensor to detect low concentrations of endotoxin). However, closely spaced AuNPs themselves could, next to coupling to the Au film, also undergo interparticle coupling. Interparticle coupling influences the scattering spectrum and therefore negatively affects measurement precision. Furthermore, high AuNP densities may also fail to yield single-particle resolution due to AuNP scattering and detector resolution. To ensure that we do not suffer from the interparticle coupling of AuNPs and to achieve single-particle resolution, we used a ratio between the ─OH- and ─COOH-terminated thiols that resulted in an average spacing between AuNPs of 3.6 ± 2.4 μm.

**Fig. 2.**
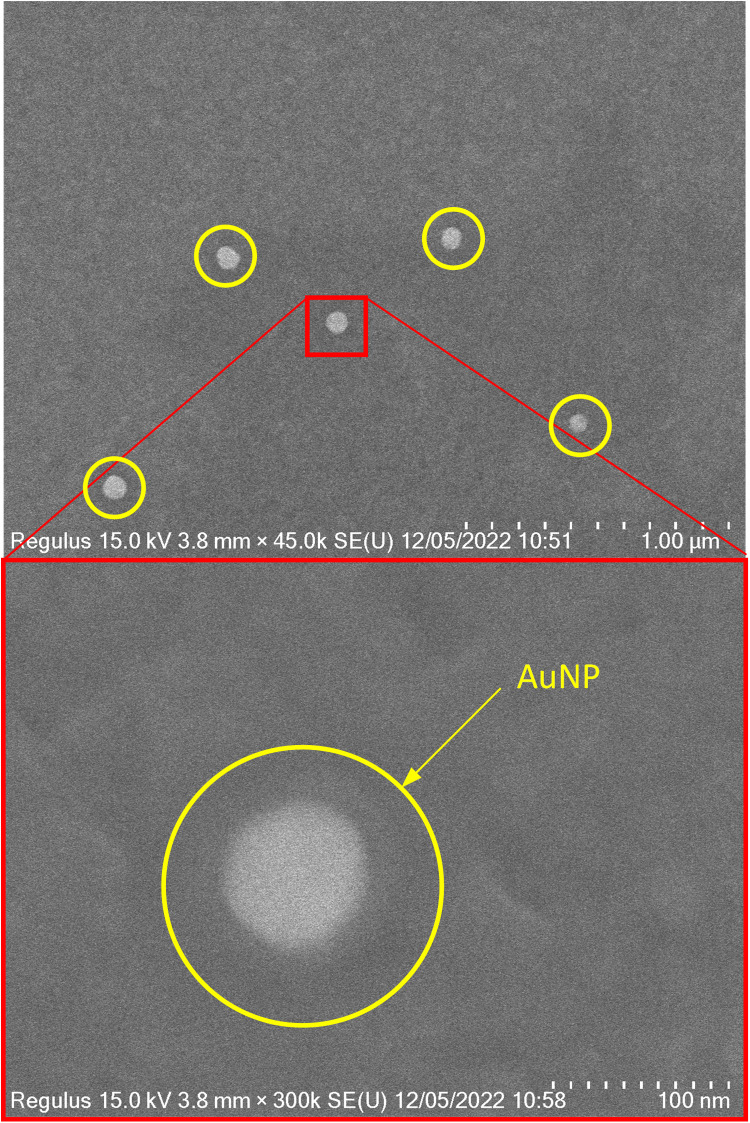
SEM images of the endotoxin aptasensor. AuNPs can be seen as bright spots with an average interparticle distance of 3.6 μm. A single particle with higher magnification is shown in the bottom image. The specific particle size appears as approximately 85 nm in diameter.

The coupling of AuNP to the AuNF via an endotoxin-specific aptamer was achieved by forming an Au─S covalent bond between the aptamer and AuNP. The formation of SAM and immobilization of aptamers were characterized by x-ray photoelectron spectroscopy (XPS; fig. S3) and quartz crystal microbalance (QCM; figs. S4 and S5). The final sensor was imaged with scanning electron microscopy (SEM; Fig. 2).

Figure S3 presents the XPS characterization of the sensor fabrication process. XPS measurements confirmed the successful covalent functionalization of the SAM layer onto the AuNF (fig. S1B). Figure S3 (B and C) further confirms the functionalization of aptamers onto the AuNF via the SAM layer.

The aptamer functionalization to SAM and the gold film–bound aptamer’s ability to bind to endotoxin were further investigated via QCM (fig. S4). Upon the formation of the SAM layer with 5.75% ─COOH and 94.25% ─OH end groups, an *N*-hydroxysuccinimide sodium salt (NHS) and 1-ethyl-3-(3-dimethylaminopropyl)carbodiimide (EDC) mixture was flown over (fig. S4, top, orange), after which RD1 aptamers were introduced into the system (fig. S4, top, green). With QCM measurements, the measured frequency change can be converted to an adsorbed mass using the Sauerbrey equation (*30*). For the RD1 aptamer immobilization step, a frequency drop of 33 Hz was observed after washing with phosphate-buffered saline (PBS). This suggests that ~50% of the ─COOH groups couple to the ─NH_2_ group of the RD1 aptamers (5.5 × 10^12^ molecules/cm^2^ as calculated by the Sauerbrey equation versus 1.1 × 10^13^ molecules/cm^2^ expected based on the ─COOH concentration on the surface). Upon introduction of the endotoxin (500 EU/ml), a frequency drop of 3 Hz, with a linear slope of −0.021 Hz/min, was observed (fig. S4, red), which means that ~41% of the aptamers bound an endotoxin (2.3 × 10^12^ molecules/cm^2^, as calculated by the Sauerbrey equation). A control group without the RD1 aptamer did not show significant differences upon exposure to endotoxin under the same flow conditions (fig. S5), demonstrating negligible nonspecific adsorption. These results suggest the functionalization of the RD1 aptamer onto the AuNF and verified the system’s ability to bind endotoxin. In addition, the SAM’s ability to passivate against the binding of the aptamers ─SH group directly to the AuNF was investigated (fig. S6). The aptamer was incubated on a sensor with SAM consisting of only ─OH groups, and no significant binding of the aptamer (─SH to AuNF) can be seen, indicating the complete coverage of the AuNF by the SAM.

AuNPs were then immobilized and passivated using 2-mercaptoethanol. This step is crucial, since it will reduce the nonspecific binding to the AuNP. The concentration of the applied 2-mercaptoethanol was optimized, as too high concentrations of 2-mercapoethanol could result in displacement of the AuNPs from the RD1 aptamer (*31*, *32*) (see Materials and Methods for details). Figure 2 presents the SEM characterization of the sensor. Individual AuNPs were distinguishable under SEM, which showed that the average interparticle distance was 3.6 ± 2.4 μm and confirmed that no particle aggregation took place. The distance between the AuNPs is several times the wavelength of the excitation light (430 to 800 nm); hence, the optical interference between particles is negligible (*33*–*38*). This makes individual resolving of the AuNPs possible with, for example, dark-field microscopy. The overall setup for the sensing can be seen on fig. S7.

### Simulation of the scattering spectrum based on Monte Carlo and dynamic simulations coupled to the plasmonic resonance

In the proposed assay, RD1 serves as a molecular ruler that controls the distance between the AuNF and the AuNP (*39*). In the absence of the target analyte (i.e., endotoxin), the aptamer is unfolded (Fig. 1A). Upon binding of RD1 to the target, the aptamer folds around the endotoxin, which results in a reduction of the average distance between AuNP and the AuNF over time. We performed Monte Carlo simulations to numerically predict the time-averaged optical signal from a single aptasensor unit with and without the presence of endotoxin(*35*,*40*). By Monte Carlo simulations, the tether composition–dependent position distribution of the 80-nm AuNP is determined. From these data, a potential energy map is created, which serves as an input to the Brownian dynamic simulations. The output of these Brownian dynamic simulations is time series of the AuNP position. The distribution of positions can be used to calculate the time-averaged plasmonic signal using the electromagnetic boundary element method (BEM) simulation. For more detailed information regarding these simulations, please consult the Supplementary Materials.

**Fig. 3.**
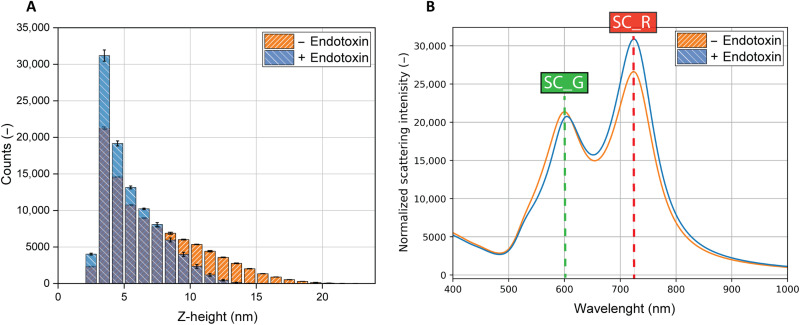
Simulated z-height and scattering spectra for the AuNP aptamer bound and unbound to an endotoxin molecule. (**A**) Time-averaged z-height of the AuNP tethered to the AuNF via the 66-nt long aptamer with its corresponding linkers with (5.40 ± 2.17 nm) and without (7.14 ± 3.6 nm) the presence of the endotoxin. The presented data of the − endotoxin is the average of three individual time traces; for the + endotoxin, two simulations per possible endotoxin length were selected (with varying endotoxin length of 2 to 4 nm; see the Supplementary Materials) and can be considered statistically significant (*P* < 0.0001) from the tethered without endotoxin. (**B**) Time-weighted scattering spectrum of a single AuNP on a AuNF using the z-height distributions as presented in (A), combined with electromagnetic BEM simulations to obtain the z-height–dependent time-averaged scattering.

Figure 3 shows the time-dependent AuNP z-height distribution and corresponding scattering spectrum. Figure 3A shows that independent of the presence of the endotoxin, the AuNP will spend most of its time close to the AuNF. The presented data of the RD1 aptamer without endotoxin are the average of three individual time traces; for the RD1 aptamer associated with the endotoxin, two simulations per possible endotoxin length were selected (with varying endotoxin length of 2 to 4 nm; see the Supplementary Materials). The intradifferences between the simulations are considered nonsignificant [*P* > > 0.05, analysis of variance (ANOVA)], while the interdifferences can be considered statistically significant (*P* < 0.0001, Student’s *t* test). In Fig. 3B, two distinct peaks can be seen, one (SC_G) in the green regime (around 600 nm) and one (SC_R) in red regime (around 720 nm), with SC_G increasing and SC_R decreasing with AuNP-AuNF distances. Although the height of the peak around 720 nm can be used for quantification of the distance between the AuNP and the AuNF, and hence the detection of the analyte, experimentally, the RG ratio = SC_R/SC_G gives a more reliable estimate of the distance since it is independent of any offset in the scattering/excitation intensity. The experimentally measured value of the scattering intensity depends on the amount of AuNPs in the spectrometer’s spot, which may vary significantly. We emphasize that this is the simulation of a single particle over time on a perfect AuNF. For ensemble measurements, several factors will influence the measured average scattering spectra, among others, the variation in AuNP size, and differences in AuNF roughness over the sensor (*41*). Furthermore, depending on the target concentration and the number of aptamers on the sensor, only a fraction of the aptamers will be bound to the endotoxin. The measured spectra will sum up all the bound and unbound states. Our simulations suggest that the higher the concentration of analyte, the higher the contribution of the 720-nm peak, and the more redshifted the average scattering spectrum will be.

### Optical sensing in ensemble mode

**Fig. 4.**
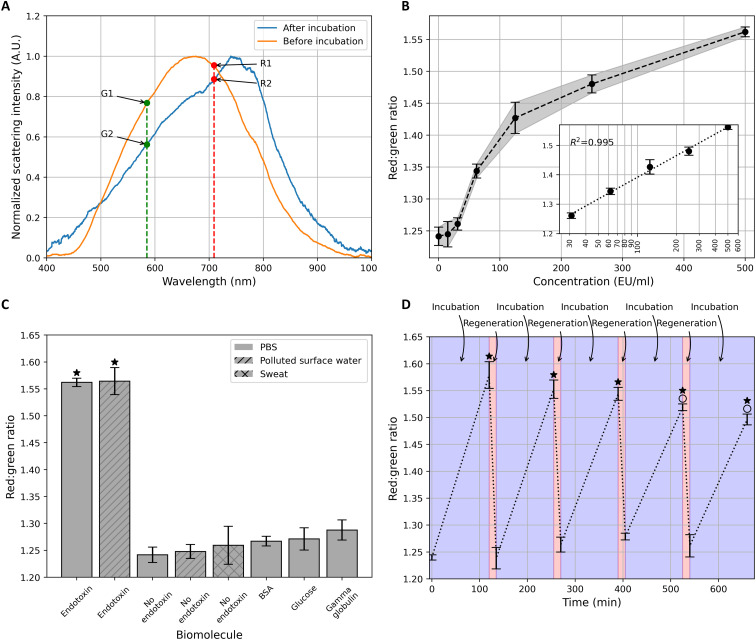
Characterization of the aptasensor. (**A**) Normalized scattering spectra before and after 2 hours of incubation with endotoxin (500 EU/ml). Before incubation: RG = R_1_/G_1_ = 1.242. After incubation: RG = R_2_/G_2_ = 1.563. The intensity of the red regime was measured at 709 nm and for the green regime at 585 nm for both spectra as shown in the figure. A.U., absorbance units. (**B**) Optical signal of the sensor upon exposure to endotoxin at various concentrations. Each point represents the average of three values, and the error bars represent the SD. The inset shows the sensitivity graph with a logarithmic scale of the *x* axis , and the dotted line shows the linear fit with an *R*^2^ of 0.995. The fitting line is *y* = 0.106*x* + 0.902. (**C**) Specificity investigation of the endotoxin sensor. PBS represents the control measurement without any biomolecule. The bar height represents the mean value of three measurements and the error bars the SD of those measurements. A one-way ANOVA analysis between PBS, bovine serum albumin (BSA), glucose, gamma globulin, human sweat, and polluted surface water groups reveals no significant differences in the absence of endotoxin. BSA, glucose, and gamma globulin concentrations were 250 μg/ml. Endotoxin concentration was 500 EU/ml (i.e., 50 ng/ml). (**D**) Regeneration of the endotoxin aptasensor. The RG ratio after five rounds of incubation (blue) between four rounds of regeneration (red) is presented (*N* = 3). The markers indicate the measured data points. The dotted line is there for visual guidance. ★*P* < 0.05 when compared with the signal without endotoxin. ^O^*P* < 0.05 when compared with the initial endotoxin incubation response.

Figure 4A presents the normalized scattering spectra measured by microspectrometry under dark-field illumination before and after 2 hours of incubation with endotoxin. The RG ratio increased from 1.242 ± 0.014 (here and below, mean ± SD) to 1.563 ± 0.008 upon exposure to endotoxin (500 EU/ml). This increase in the RG ratio was in accordance with the simulation results (Fig. 3). The “green” value of the scattering intensity was taken at 585-nm wavelength and the “red” value at 709-nm wavelength. The same wavelengths are used for the calculations of RG for the rest of this study. The chosen red (709 nm) and green (585 nm) wavelengths are slightly off the predicted 720 and 600 nm by the simulations but empirically produced better results for the RG ratio. A possible explanation for this behavior could be that the excitation light intensity is stronger at these chosen values due to the emission spectrum of the lamp.

### Analytical sensitivity

The analytical sensitivity can be defined as the slope of the calibration curve (Figure 4B inset). Figure 4B presents the RG ratio after incubating our sensor with seven different endotoxin concentrations between 0 and 500 EU/ml for a duration of 2 hours. The RG ratio scales linearly with the logarithm of the concentration with a slope of 0.106 RG/log (EU/ml). The results show that our detection limit in the ensemble mode lies between 15 and 31 EU/ml. This indicates the promising application of our sensor in the food and pharmaceutical industry, e.g., according to the U.S. Food and Drug Administration guidelines, parenteral drugs such as cyanocobalamin injections have an endotoxin limit of 350 EU/ml, well above the limit of detection of our sensor (*42*). Furthermore, the strong linearity in the logarithm scales demonstrates the high reliability of our sensor.

### Endotoxin detection under polluted surface water

The functionality of our sensor under polluted surface water was demonstrated in Fig. 4C. The RG ratio increased from a mean of 1.25 ± 0.01 to 1.56 ± 0.03 upon incubation with endotoxin in polluted surface water. Furthermore, incubation with polluted surface water showed no significant difference when compared with PBS incubation, both under the conditions with and without endotoxin. This shows the promising real-life applicability of our sensor in endotoxin sensing.

### Specificity

To investigate the specificity of our sensor, three different competitor biomolecules found in endotoxin applications were tested, namely, bovine serum albumin (BSA), glucose, and gamma globulin (250 μg/ml) using PBS as a solvent. Note that the concentrations of the competitor molecules were tested at weight concentrations 5000 times higher than that of endotoxin to amplify the nonspecificity and provide accurate information on the specificity of our sensor. As shown in Fig. 4C, no significant increase in the RG ratio (*P* > 0.05) can be seen for any of the competitors compared with the PBS control. In addition, the specificity of this sensor for two other real-life relevant fluids was tested, namely, human sweat and polluted surface water. For both cases, no significant increase in the RG ratio was observed in the absence of endotoxin.

### Regeneration and reusability

Figure 4D demonstrates the regenerability and reusability of this platform. The RG ratio increased by 0.339 ± 0.024 after 2 hours of incubation with endotoxin, after which the sensor was regenerated upon incubation in Milli-Q water at 95°C for 15 min. This temperature is above the DNA aptamer’s melting point, which breaks the aptamer-aptamer and the aptamer-endotoxin hydrogen bonds, resulting in the unfolding of the aptamer and releasing endotoxin. A second incubation round with endotoxin raised the RG ratio by 0.273 ± 0.017, which was successfully regenerated by incubation in Milli-Q water. Third, fourth, and fifth cycles were successfully demonstrated subsequently. The first three RG values after incubation with endotoxin showed no significant differences (*P* > 0.05), indicating promising sensor repeatability and reproducibility after regeneration. Although the endotoxin response after the last two regeneration events showed significant differences from the initial endotoxin response, the RG ratio before and after endotoxin incubation remained statistically significant. This simple regeneration method by heating enables repeated measurements to be conducted from a single device, which empowers measurements at regular intervals in a time- and cost-effective manner. The optimization of the regeneration process will be investigated in the future, by tuning the temperature, performing the regeneration under flow to remove the released endotoxin and tune the pH of the solvent as reported in other studies (*43*).

### Stability

Figure S10 shows sensitivity of decay versus time. Sensors were prepared on day 0 and stored in ambient air and room temperature. No significant difference in the RG ratio (*P* > 0.05) was seen up to 7 days of storage, indicating promising stability when compared with the conventional LAL method, in which refrigeration is required.

### Single-particle resolution imaging

**Fig. 5.**
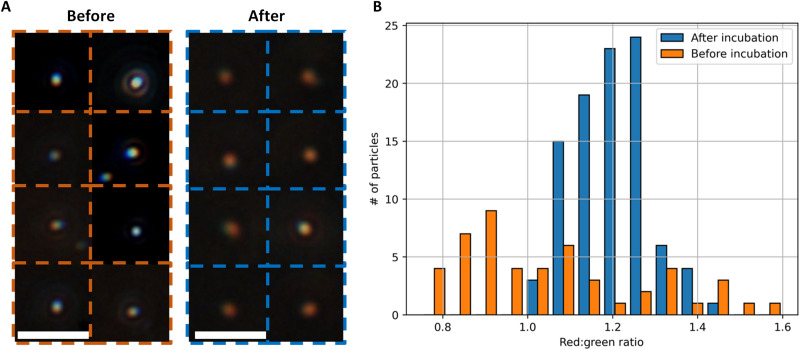
Endotoxin sensing with single-particle resolution. (**A**) Dark-field images of AuNPs before (left) and after (right) incubation with endotoxin. White scale bars, 10 μm. The left and right images are separate frames and not the same particles. (**B**) RG ratio of individual AuNPs, analyzed by dark-field microscopy (*N* = 145 particles).

In this section, we present the detection of endotoxin at single-particle resolution. The scattering cross section of individual nanoparticles is large enough to be visible via optical microscopy under dark-field illumination. Figure 5A presents the dark-field images of AuNPs captured with a color camera at ×40 magnification before and after incubation with endotoxin (500 EU/ml). A clear redshift can be observed after endotoxin exposure.

Figure 5B presents the RG ratio of individual particles before and after incubation with endotoxin (500 EU/ml). The RG ratio was determined for each individual particle by calculating the average intensity of the red channel of an individual particle over the average intensity of the green channel of an individual particle. Data from a total of 145 particles are presented in Figure 5B. As expected, a clear increase in the RG value was observed. By monitoring the RG values of single particles, our platform shows the ability of endotoxin sensing with single-particle resolution.

## DISCUSSION

We have demonstrated the optical detection of endotoxin using an aptasensor based on surface plasmon resonance. Sensor fabrication was verified by XPS, QCM, and SEM analyses. The sensor has a linear response with the logarithm of the concentration of endotoxin and a limit of detection relevant to the food and pharmaceutical industry. In addition, the sensor is highly selective toward endotoxin, and a negligible response is observed when incubated with competing biomolecules and with relevant biosamples. The proposed sensor can enable single-molecule detection of endotoxin by monitoring the color of individual particles under dark-field microscopy. While these results are promising, further studies are required to investigate the sensor’s response toward endotoxin under industrial-related processing mixtures. In addition, further engineering of a continuous platform that enables real-time tracking of AuNP responses will be required to realize the promising potential of this platform in single-molecule sensing. Future work could focus on the optimization of the analytical sensitivity by playing with the AuNP density on the sensor. The final, optimized density will highly depend on the measurement mode (ensemble or single particle) and the detector’s resolution and other microscope settings, such as the objective magnification. In addition, the regeneration process will also be optimized. In summary, this platform shows high selectivity, repeatability, and reliability toward endotoxin, with the promising potential of single-molecule detection. The same sensing principle can be applied to other biomolecules by exchanging the endotoxin selective aptamer with one selective toward the molecule of interest, making this a highly versatile platform.

## MATERIALS AND METHODS

To investigate our hypothesis, the same sensing setup will be tested in QCM and microspectroscopy (optical sensing).

### Sensor preparation

The preparation of optical sensing chips was as follows (fig. S1, A to F). Starting with fused silica wafers of 2.5 cm in diameter (Microchemicals GmbH), 5 nm of chromium and 45 nm of gold were evaporated on the wafers using a Temescal FC-2000. The chromium was used as an adhesion layer between the AuNF and the substrate. The thickness of the AuNF was chosen on the basis of the work of Armstrong *et al*. (*44*). Two linkers with different end functional groups were used to form a SAM. Specifically, 2 mM HSC_11_EG_6_OCH_2_COOH and 2 mM HSC_11_EG_5_OH (ProChimia Surfaces) were dissolved in ethanol (99.5%; Sigma-Aldrich) and incubated on the sensor overnight. The aptamer RD1 (*20*) was chosen for its affinity against endotoxin (shown in italics in the sequence below), and two 15–nucleotide (nt)–long sequences were added to either end of the aptamer to increase its overall length. The two sequences are noncomplementary with each other and not self-complementary, so they are expected to remain unfolded, and they were designed with the use of the EGNAS algorithm (*45*). The final aptamer sequence is the following 5′-AAGAAACAGTGAGGA*GTCGAATGCTCTGCCTGGAAGAGTTGTTAGCAGGGA*ACAGGAAAGAGTGGT-3′ (Merck). The aptamer has an amine group modification at the 5′ end and a thiol group modification at the 3′ end. The aptamers were heated above their melting point at 95°C for 15 min to ensure that no double-stranded DNA was present. Once the SAM was formed, the carboxylic groups were activated by 100 mM NHS and 100 mM EDC in PBS (Sigma-Aldrich), forming an NHS ester. With the incubation of the 0.67 μM aptamer in PBS, the NHS ester on the linkers reacted with the amine group on the 5′ end of the aptamer, binding it to the AuNF (fig. S1D). Next, a solution of AuNP (BBI Solutions) of 80 nm in diameter was incubated with the thiol group of the aptamer, tethering the AuNPs to the AuNF. Last, an incubation of 1 μM 2-mercaptoethanol ensures the passivation of the AuNP against any nonspecific binding.

### Simulation of optical signals

AuNP z-height distributions were calculated by combining Monte Carlo simulations with Brownian dynamic and electromagnetic BEM simulations as described elsewhere (*46*). A more detailed description of the simulation methods can be found in the Supplementary Materials. In short, the entire tether (consisting of aptamer and corresponding linkers) is simulated as a composite worm-like chain. In the first step, 10^5^ individual chains are generated, taking several boundary conditions into consideration. The AuNP position of these chains is used to generate an energy contour and force gradient using inverse Boltzmann statistics. This energy map is the base of the Brownian dynamic simulations, where the z-height position over time is determined with a Δ*t* of 10^−8^ s. The positions over time are used as an input of the electromagnetic BEM simulations, which enables us to predict the time-averaged scattering spectra for aptasensors bound to endotoxin and “free” aptasensors. All calculations regarding the simulation of the optical signals were performed in MATLAB R2021a.

### XPS characterization

To evaluate the chemical element, present on the surface and its state, XPS (Thermo Fisher Scientific Nexsa) measurements were carried out using a monochromatic Al Kα radiation source with a spot size of 50 μm. For each sample, two types of scans were conducted. The survey scans (pass energy of 140 eV and 40 scans) were performed to obtain the surface element. To evaluate the elements (nitrogen and sulfur), more specific scans were conducted with higher resolution (pass energy of 50 eV and 80 scans). The resulting surface charge was compensated by a magnetic charge compensation system, and the energy scale was calibrated against the Au4f_7/2_ peak (84.0 eV). The spectra were further deconvoluted using a postprocessing CASA-XPS software with Shirley background and a mixed Gaussian-Lorentzian peak fitting along with a minimum number of peaks consistent with a reasonable fit to the spectral data and the molecular structure.

### SEM imaging

Field emission SEM (FE-SEM) was used to obtain the surface topology (Hitachi Regulus SU8230). The samples were placed on the microscope holder and adhered to a carbon tape. It was then loaded into the FE-SEM chamber, with a working distance of 3.8 mm, an accelerating voltage of 15 kV, and an emission current of 700 nA. The reading from secondary electron detectors was then used to obtain the topological information.

### Optical sensing characterization

The scattering spectra were recorded with a Flame VIS-NIR spectrometer (Ocean Insight) coupled to an Axiovert100M (Zeiss) microscope under dark-field illumination, as shown in Fig. 1B. The data were recorded via OceanView (Ocean Insight) software and analyzed in MATLAB R2020b. A stock solution containing endotoxin (4000 EU/ml or 400 ng/ml) (LPSs from *Escherichia coli* O111:B4; L2630, Sigma-Aldrich) in endotoxin-free water was prepared according to the protocol from its supplier. Further dilution was performed to make final incubation solution containing 500, 250, 125, 62, and 31 EU/ml, respectively, in Milli-Q water. Meanwhile, human sweat was collected upon consent. A 3M tegaderm and gauze bandage were placed on the healthy subject, and sweat was collected upon exercise. Polluted surface water was collected from the canals of our campus (Delft, The Netherlands). Impurities were removed by centrifugation at 5000*g* for 30 min. A bare AuNF was used as background, measurements from which were subtracted from the aptasensor results. In addition, the aptasensor measurements were further normalized with respect to the excitation light.

### QCM characterization

Characterization from the QCM was conducted using a QE401 electronics unit with a QFM401 flow module from Biolin Scientific. The same protocol was used for the QCM sensors performing the SAM formation outside the QCM and all the subsequent steps in the flow module. The data were recorded via QSoft (Biolin Scientific) software and analyzed in MATLAB R2020b. The dilution of a stock solution containing endotoxin (4000 EU/ml) was performed to make a final solution containing 500 EU/ml in PBS.

### Statistical analysis

Statistical analysis between two groups was performed by a Student’s *t* test. Statistical analysis of three or more groups was performed by one-way ANOVA. Statistical significance was determined when the *P* value was <0.05.

## Supplementary Materials

### This PDF file includes:

Supplementary Text Figs. S1 to S10 References
