# Supplementary material for: An optical aptasensor for real-time quantification of endotoxin: From ensemble to single-molecule resolution
Source: Sci Adv. 2023 Feb 8;9(6):eadf5509. doi: 10.1126/sciadv.adf5509 (PMC9908015; doi:10.1126/sciadv.adf5509)
Supplement: Supplementary file 1 — Supplementary Text Figs. S1 to S10 References [file sciadv.adf5509_sm.pdf]

Science Advances
AAAS

Supplementary Materials for

An optical aptasensor for real-time quantification of endotoxin: From ensemble to single-molecule resolution

Pancheng Zhu *et al.*

Corresponding author: Alina Y. Rwei, a.y.rwei@tudelft.nl; Loes I. Segerink, l.i.segerink@utwente.nl

Sci. Adv. **9**, eadf5509 (2023)
DOI: 10.1126/sciadv.adf5509

This PDF file includes:

Supplementary Text
Figs. S1 to S10
References

Supplementary Text

Effect of SAM layer composition on density of particles

Fig. S2 shows the dark field images of the assembled sensor with varied composition of –COOH and –OH end groups in the SAM layer. We show that the AuNP functionalization density decreases with decreasing –COOH content.

XPS characterization

Fig. S3A presents results from XPS on the binding energy (BE) of the chemical element sulfur to verify the covalent attachment of the thiol-containing SAM layer on the AuNF (shown in Fig. S1B). A low pass energy (50 eV) was used to reduce the X-ray damage on sulfur. Fig. S3A demonstrates that the SAM-functionalized AuNF exhibited two peaks corresponding to S2p BE spectra. This indicates the presence of sulfur on the surface of the AuNF. To gain further insight regarding whether the sulfur was bound, the peaks were then fitted based on the following sulfur states: (i) an unbound thiol group (or weakly physiosorbed) typically shows a BE signal of $S2p_{3/2}$ centered between 163 and 164 eV; and (ii) when the thiol is bound to Au (or strongly chemisorbed), the BE decreases to around 162eV (*47, 48*). This analysis resulted in the observation of 4 peaks; where two peaks represent the peaks of unbound thiol with its spin-orbit splitting ($S2p_{3/2}$ Un and $S2p_{1/2}$ Un, Fig. S3A) and the other two of bound thiol ($S2p_{3/2}$ B and $S2p_{1/2}$ B, Fig. S3A). Interestingly, the area under the curve indicates a 49 %: 51 % ratio of bound to unbound thiol, despite careful rinsing with ethanol and water. This presence of unbound thiol upon chemical functionalization is consistent with previous studies (*49*). These results indicate the successful covalent functionalization of the SAM layer onto the AuNF.

The sensor base was then incubated with NHS/EDC and the aptamer to form bonds between the aptamers and SAM. This bonding was evaluated by looking into the nitrogen BE signal, as the aptamer contains N in their aromatic groups (e.g., pyridine, adenine) and amine, both are indicated with the N1s BE peak at around 400 eV (*50, 51*). Figs. S3B&C present the N1s BE peak before and after incubation respectively. While no observable peak was present prior to the introduction of the aptamer (Fig. S3B), indicating the absence of N1s, a clear peak at 400 eV was observed after the system was functionalized with aptamer (Fig. S3C). This indicates the presence of N1s, attributed to the nitrogen-containing aptamer. These results show the successful functionalization of aptamers onto AuNF via the SAM layer.

Quartz crystal microbalance (QCM)

QCM was used for the investigation of the functionality of the aptamers and the self-assembled monolayer (SAM). On Fig. S4 the preparation of the sensor and the incubation with endotoxin can be seen. The experiment starts with the incubation of SAM on an QCM gold coated sensor. Once a stable frequency is seen while flowing PBS the data is recorded. The EDC:NHS (orange) reacts with the -COOH groups of the (SAM's) linkers creating a NHS ester. The added mass of the NHS esters can be seen by drop in frequency on Fig. S4-top. After a rinse step with PBS the aptamer is incubated. The amine group at the 5'end reacts with the NHS ester. The added mass of the aptamer results in another drop in the frequency. After a thorough rinse with PBS, 500EU/ml of endotoxin is introduced. There is a linear drop of frequency with a slope of -0.021 Hz/min in the frequency showing successful capturing of endotoxin by the aptamer.

On Fig. S5, a similar experiment is presented but in this case no aptamer is introduced. Similar to before, EDC:NHS results in a drop of the sensor's frequency indicating successful SAM immobilization. This time the introduction of endotoxin shows no significant change in frequency, indicating that a) the SAM protects against non-specific binding of endotoxin on the sensor and b) the binding seen on Fig. S4 was due to the capturing of endotoxin by the aptamer.

Plasmonic resonance theory

The free electron cloud of metal nanoparticles can be displaced from its equilibrium position in the presence of an electromagnetic field (i.e., light), creating net and opposite charges on either side of the nanoparticle (Fig. S8). These opposite charges apply a restoring force on each other. The external force applied by light and the restoring force form an oscillating system with a specific resonance frequency. This oscillation of the free electron cloud of metal nanoparticles is called plasmon resonance (*33, 34*). When the wavelength of the light is much larger than the particle size then the electric field inside the particle is uniform and it behaves as a dipole, inducing an electric field around it which is in phase with the external electric field (*34*). The interaction of the dipole's electric field with the external results in scattering of light with maximum scattering intensity at the plasmon resonance frequency of the nanoparticle.

When two metal nanoparticles are in close proximity the electric field of the dipoles interact with each other resulting in their plasmonic coupling. The two dipoles can be coupled perpendicularly, parallelly or both depending on the polarization of the light. In our case of unpolarized light both types of coupling are present. In case of parallel (longitudinal) coupling there is a constructive interference of the electric field of the two dipoles. The two-dipole system has lower resonance frequency resulting in a red shift in the maximum scattering wavelength compared to an individual nanoparticle. In contrast in the perpendicular (transversal) coupling there is destructive interference between the electric fields, which results in a higher resonance frequency of the system compared to individual nanoparticles hence a blue shift in the maximum scattered wavelength is seen (*33-38*). Similar plasmon coupling is also occurring between a metal nanoparticle and a metal film. The nanoparticle's dipole induces an electric field to the local free electrons in the film. The nanoparticles dipole is plasmonically coupled (longitudinal and transversal) to its mirror image in the film as shown on Fig. S8 (*37*, *39*, *44*). The plasmonic resonance frequency and hence the scattering light wavelength depends on the particle material, particle size, film thickness, distance between particle and film, and angle of incident light (*19*, *37*). Different angles of incident lights change the ratio between longitudinal and transversal coupling.

Monte Carlo Simulations

Monte Carlo simulations were performed similar to published earlier by van Dongen *et al* (*40*). The tether AuNP complex is simulated as a composite worm-like chain (WLC) (*52-53*) with varying compositions, depending on the state. The chain building process is repeated until 10^5 valid chain states are generated with the set contour length. Chains were considered valid if no tether-surface, tether-AUNP, or AuNP surface collisions occurred during the chain building process. Each WLC component (Propane, hexane, ssDNA and endotoxin) had their own persistence length (P), contour length (L) and discretization length worm-like chain (l_s). The different properties can be found in Tab. S1.

Tab. S1. Properties of the tether Worm-Like Chains used in this paper.

	**Parameter**	**Value**	**Refs./ remarks**
Endotoxin	l_s	1 nm	Length scale single peptide unit (*54-55*)
	P	1 nm	(*55-56*)
	L	2-4 nm	Model choice, based on (*57*)
ssDNA	l_s	0.1 nm	Model choice. (1/20 of the persistence length, commonly chosen as demonstrated by (*58*))
	P	2 nm	(*59*)
	L	15 nt on top and bottom of endotoxin,	Model choice.

		or 66 nt (without endotoxin)	
Hexane	l_s	0.03 nm	Model choice. (1/50 of the persistence length, commonly chosen)
	P	0.5 nm	(*60*)
	L	0.63 nm	Calculated estimate based on the notion that each C-C bond results in a contour length of approximately 1.26Å.
Propane	l_s	0.03 nm	Model choice. (1/50 of the persistence length, commonly chosen)
	P	0.5 nm	(*60*)
	L	0.25 nm	Calculated estimate based on the notion that each C-C bond results in a contour length of approximately 1.26Å.

A challenge in these specific Monte Carlo simulations is the simulation of the endotoxin interacting with the ssDNA. Very little is known about the precise nature of the interaction between the ssDNA aptamer and the endotoxin. We therefore decided to approximate the ssDNA interaction with the endotoxin by taking the average size of the endotoxin we can find in literature (*61*) Which varies between ~2 and 4 nm. The segment length was decided to the length scale of a single peptide unit, as well as the persistence length we could find for proteins in literature (both around ~1 nm) (*55-56*).

Following the Monte Carlo simulations, 2D Brownian dynamic simulations were performed in MATLAB (*40*). Due to the relative size of the bead compared to the contour length of the entire WLC chain, we assumed that the hydrodynamic properties of the AuNP dominate the overall motion. Because the AuNP spends most of its time close to the AuNF, we implemented the hydrodynamic effects the AuNP experiences by the use of Faxen's law, where z=0 is set at the interface between Au film and the Self-assembled monolayer.

The resulting time series we obtain from the Brownian dynamics simulations has a time resolution of 10^{-8} seconds. We integrated the results to obtain an integration time of 10^{-6} seconds.

<u>Boundary element method (BEM) simulations</u>

Similar to earlier published work (*40*), a free MATLAB toolbox called the MNPBEM simulation toolbox was used to simulate the z-height-dependent optical response of the AuNPs numerically. The simulated system consists of a sensor surface with a layered structure on which a AuNP is immobilized via ssDNA tethers. The layer structure of the sensor consists of a 1 mm thick fused silica wafer with a refractive index of 1.51, which is covered by a thin chromium (*62*) layer of 5 nm thick to ensure good adhesion of the 45 nm thick gold layer that is placed on top. On top of the AuNF, a SAM of decane (R.I.=1.405 (*63*)) is placed, to which polyethylene glycol (PEG) linkers (R.I.=1.37 (*64*)) are linked. The PEG linkers are connected to DNA tethers. The ssDNA tethers are ignored in the numerical simulation due to their relatively small volume compared to the other layers described and, therefore, their negligible effect on the plasmonic properties of the AuNP. The AuNP has a diameter of 80 nm. The sensor is placed in a 0.10 mM phosphate buffered saline (PBS) solution, which has a refractive index of 1.338 (*65*).

<u>Normalization with the spectrum of the excitation light.</u>

As we can see from Fig. S9, the lamp of the microscope does not produce light equally at every wavelength. Therefore, we need to calculate proportion factors (pf) for each wavelength, to take into account how efficient that wavelength of light is produced by lamp. The intensity of the measured spectra of the sensor in dark-field should be normalized by dividing intensity at each wavelength by the pf of each wavelength. For example, the measured intensity at 483 nm will be

divided by 0.5 to take into account that this light is produced 50 % less efficient by the lamp. One problem that comes is that in the edges of the pf curve, we have pf = 0 and we need to avoid division by zero. To avoid that, we just shift the curve slightly upwards (+0.4) and normalize again between 0 and 1. The shifted proportion factor is what we actually use to divide the measured intensities of the sensor.

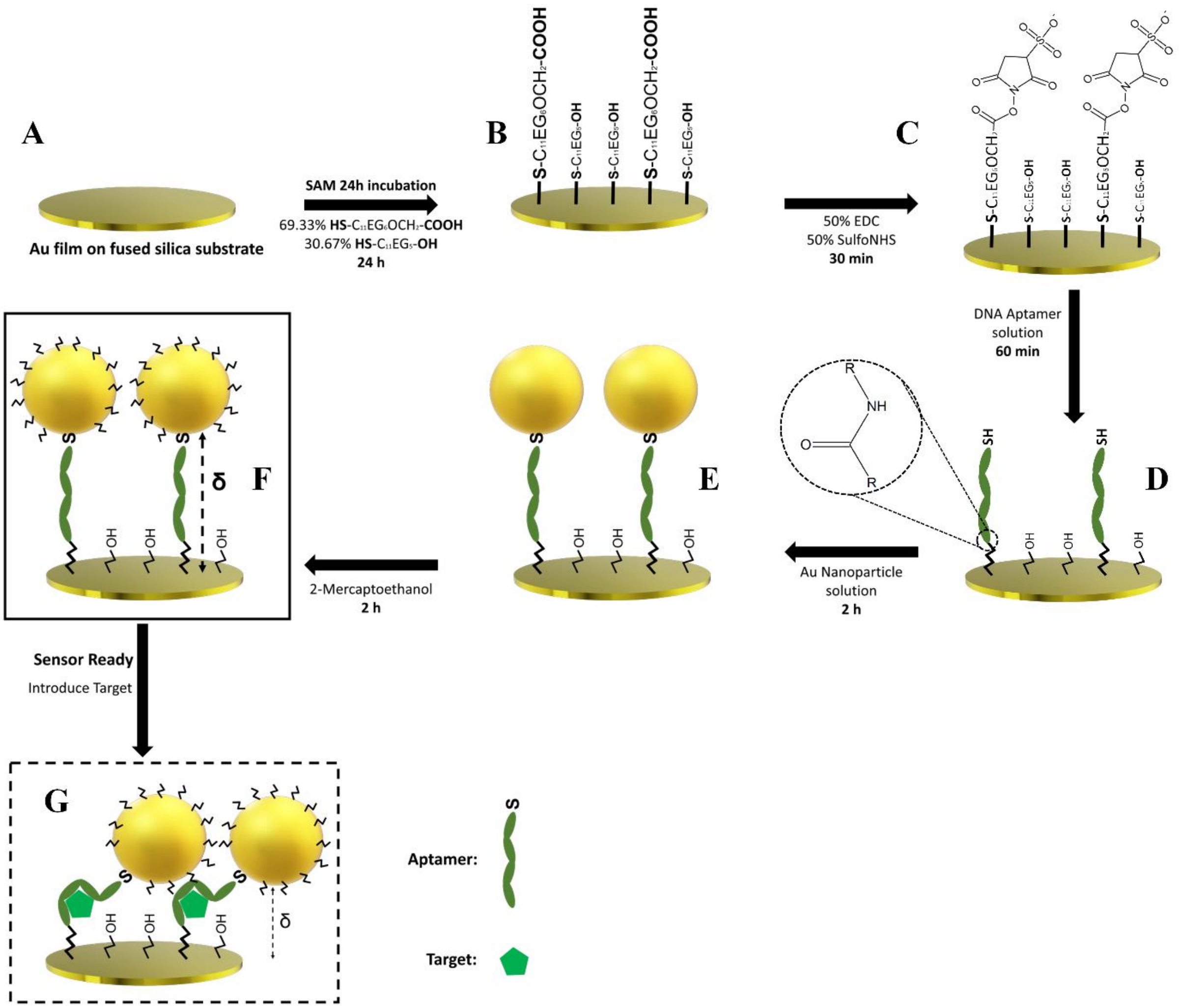

Fig. S1. Schematic of the sensor preparation process. (**A**) 45 nm Au with 5 nm Cr film is deposited on a fused silica wafer. (**B**) Self-assembled monolayer consisting of a mixture with $HSC_{11}EG_6OCH_2COOH$ and $HSC_{11}EG_5OH$ linkers. The linkers are immobilized on gold via a thiol reaction. (**C**) Surface activation via the NHS: EDC reaction. An NHS ester is formed on the COOH group of the linkers. (**D**) Functionalization of aptamer. The amine group on the 5' end of the aptamer reacts with the NHS ester formed on the COOH group of the linkers. (**E**) AuNP is bound to the thiol group at the 3' end of the aptamer. (**F**) Mercaptoethanol creates a passivation layer around the AuNP for protection against the non-specific binding of biomolecules. (**G**) The aptamer undergoes a conformational change upon binding to the target, which reduces the distance between AuNP and gold film.

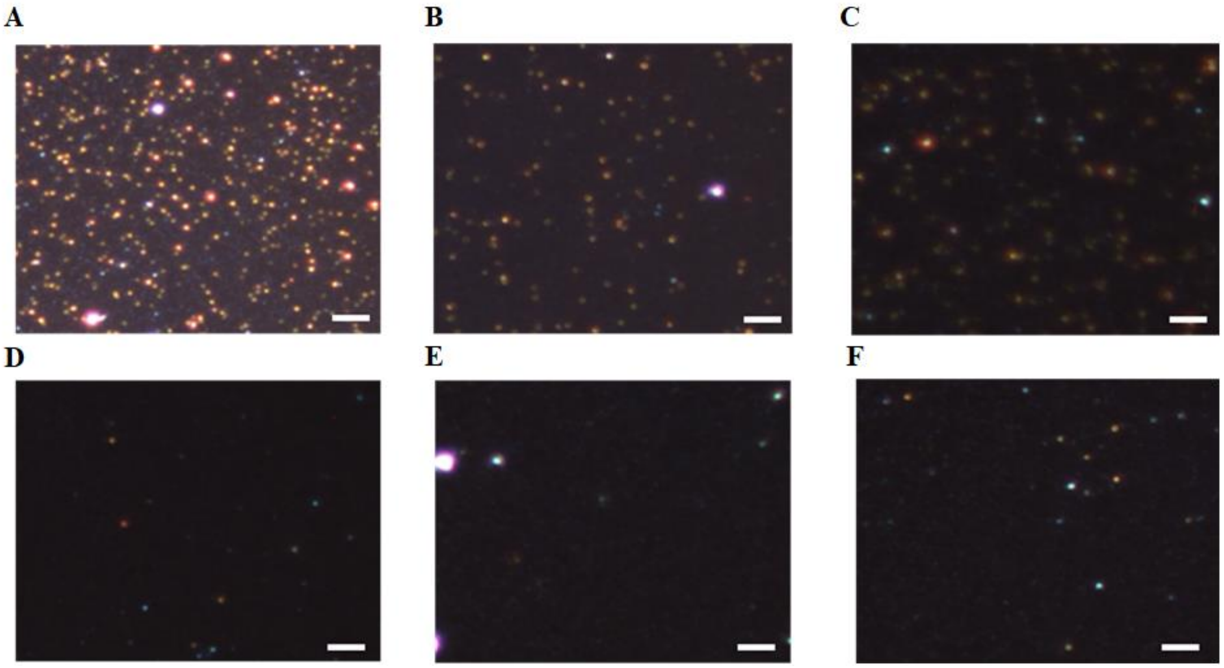

Fig. S2. Darkfield microscopy of AuNPs at different carboxyl: hydroxyl-terminated thiol ratios. (A) 30% -COOH, **(B)** 10% -COOH, (**C)** 5% -COOH, **(D)** 1% -COOH, **(E)** 0.1% -COOH, **(F)** 0.01% -COOH. Scale bar equals 5 µm.

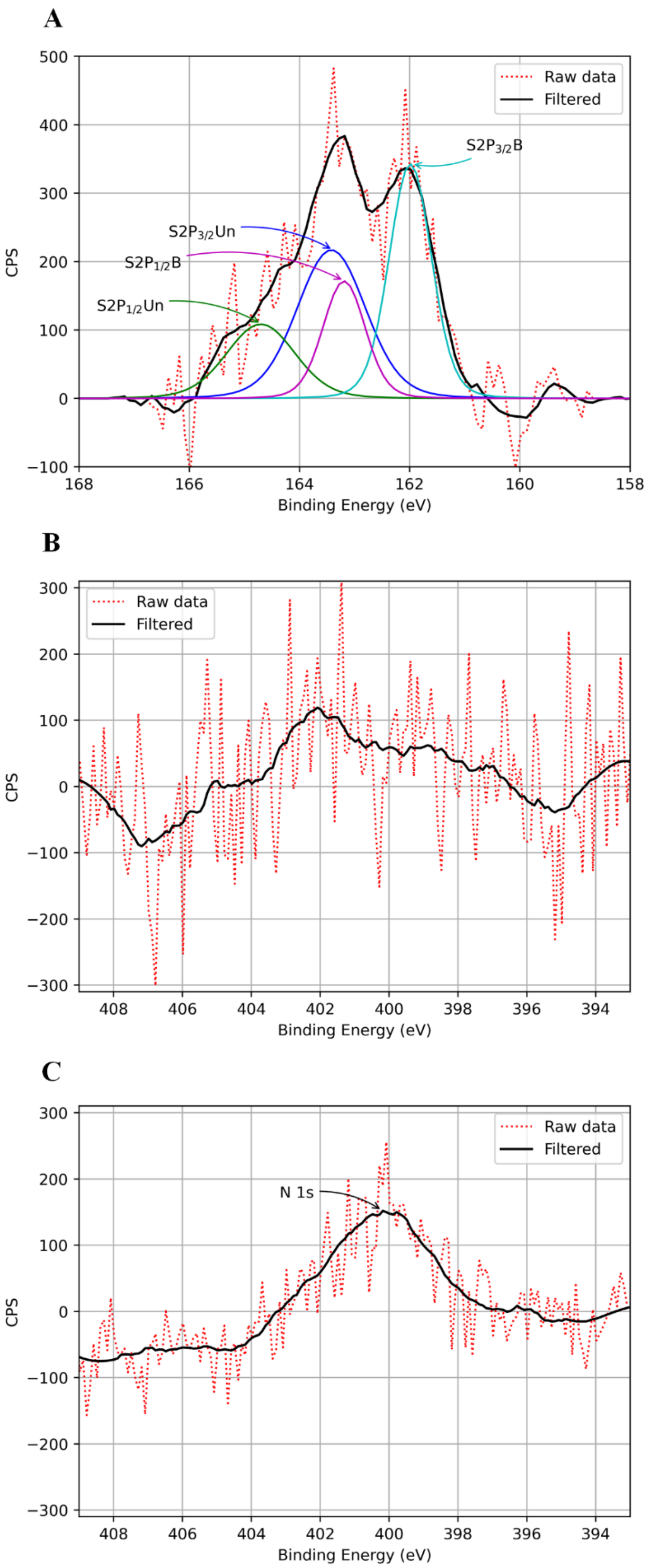

Fig. S3. XPS analysis. (**A**) S2p XPS Core level spectra of the sensors after incubation with the mixture solution of $HSC_{11}EG_6OCH_2COOH$ and $HSC_{11}EG_5OH$. (**B**) N1s XPS Core level spectra of the sensors after incubation with the mixture solution of $HSC_{11}EG_6OCH_2COOH$ and $HSC_{11}EG_5OH$. (**C**) N1s XPS Core level spectra of the sensors after incubation with EDC/NHS and RD1 aptamer. CPS = counts per second.

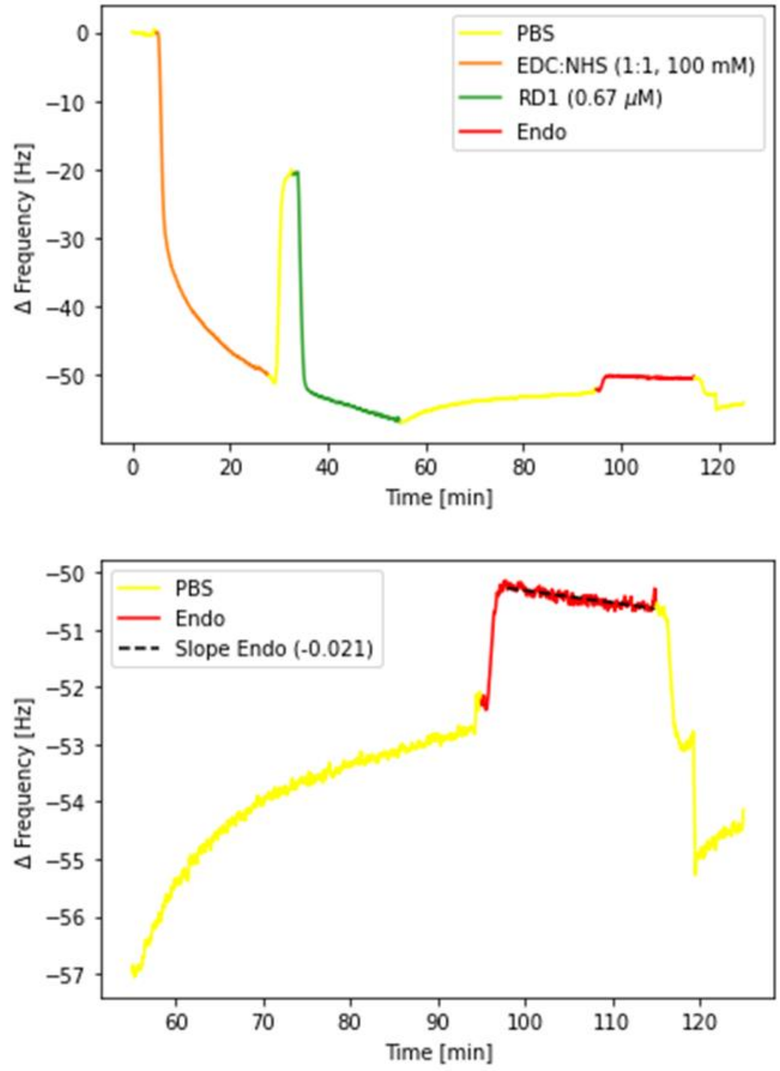

Fig. S4. Preparation of the sensor with aptamer immobilization and incubation with 500 EU/ml of endotoxin. Top-overview. Bottom-zoomed view of the endotoxin incubation. Endo = Endotoxin.

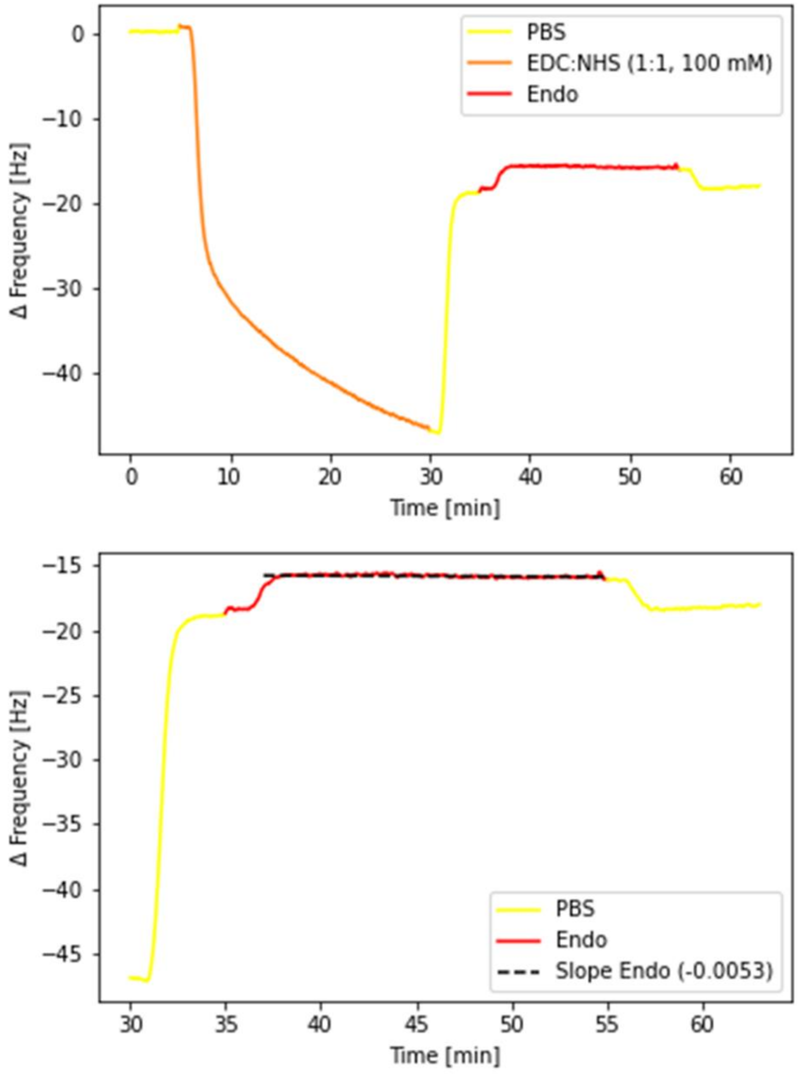

Fig. S5. Incubation of 500EU/ml of endotoxin on the sensor without immobilized aptamer. Top-overview. Bottom-zoomed view of the endotoxin incubation. Endo= Endotoxin.

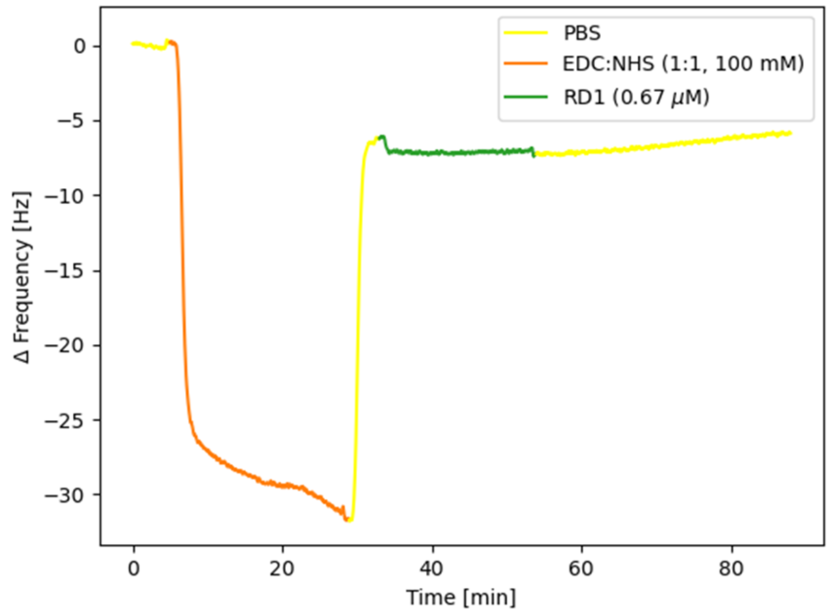

Fig. S6. Investigation on the passivation of the SAM against the aptamer -SH group binding on the AuNF. RD1 aptamer was incubated on a sensor with SAM with only -OH groups. No significant binding of aptamer can be seen, indicating the complete coverage of the AuNF by the SAM.

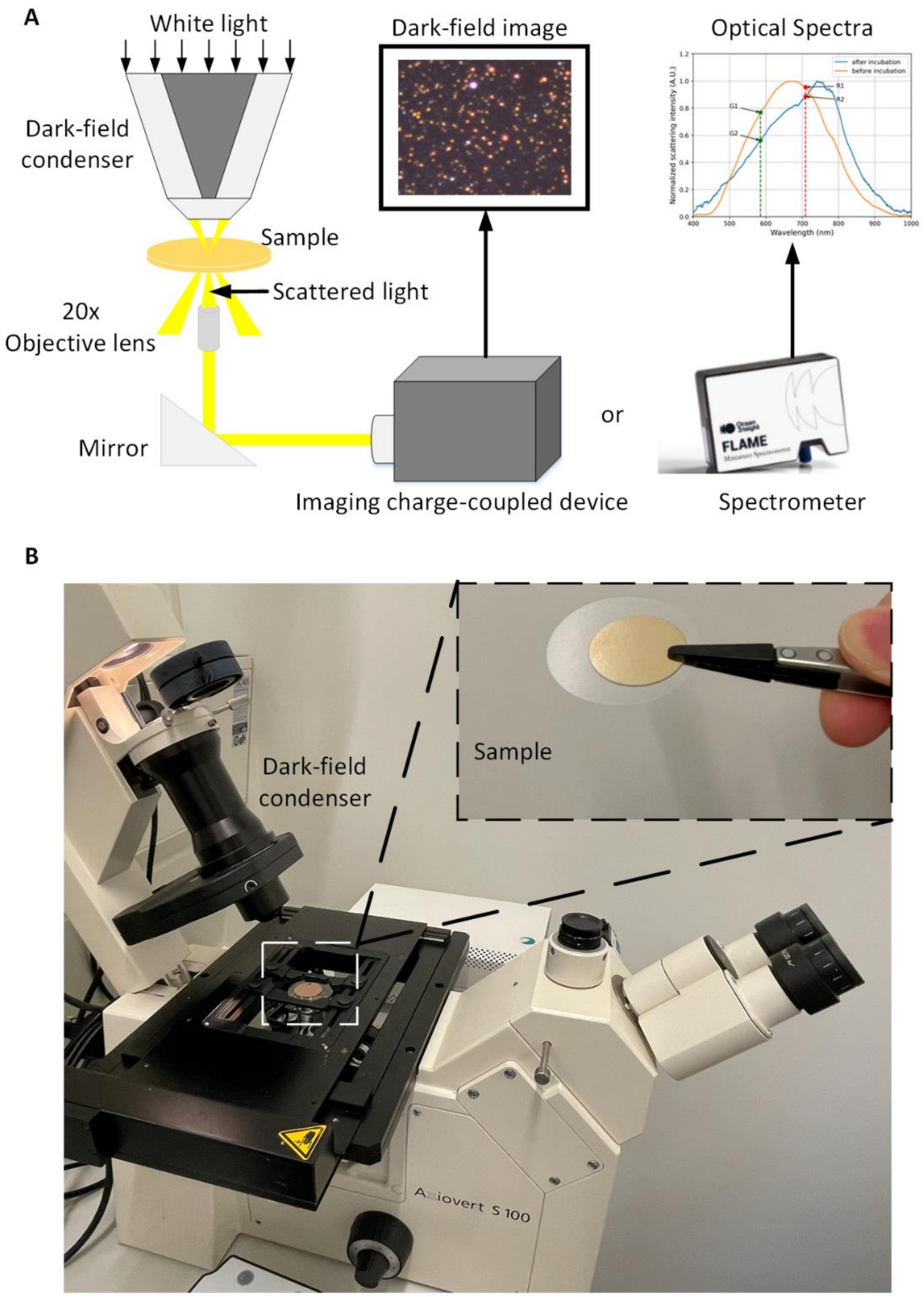

Fig. S7. Experimental setup and operating principle of the sensor. (A) Scattered light from the sensor sample is collected through an objective lens to a charge-coupled camera (for single-particle mode) or a spectrometer (for ensemble mode). (B) A photograph of the microscope setup and the sensor chip.

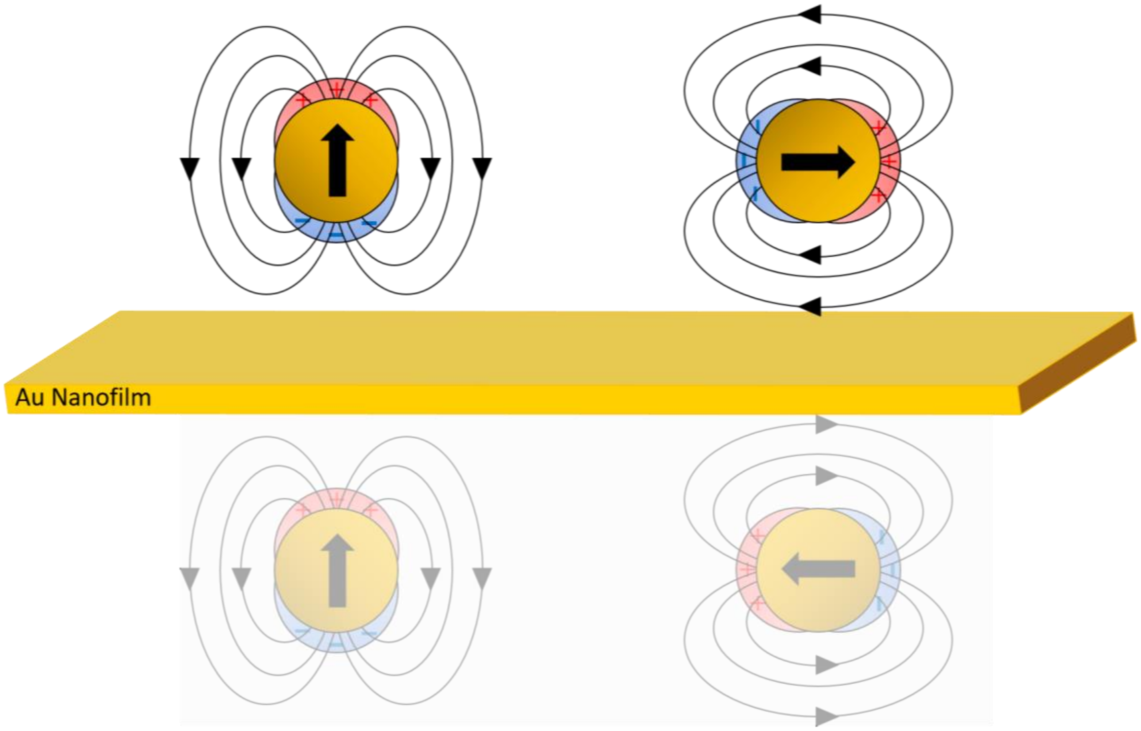

Fig. S8. Plasmonic coupling between gold nanoparticles and their mirror image in AuNF. Left-longitudinal coupling: Constructive interference results in lower plasmonic resonance frequency. Right-Transversal coupling: Destructive interference results in higher plasmonic resonance frequency.

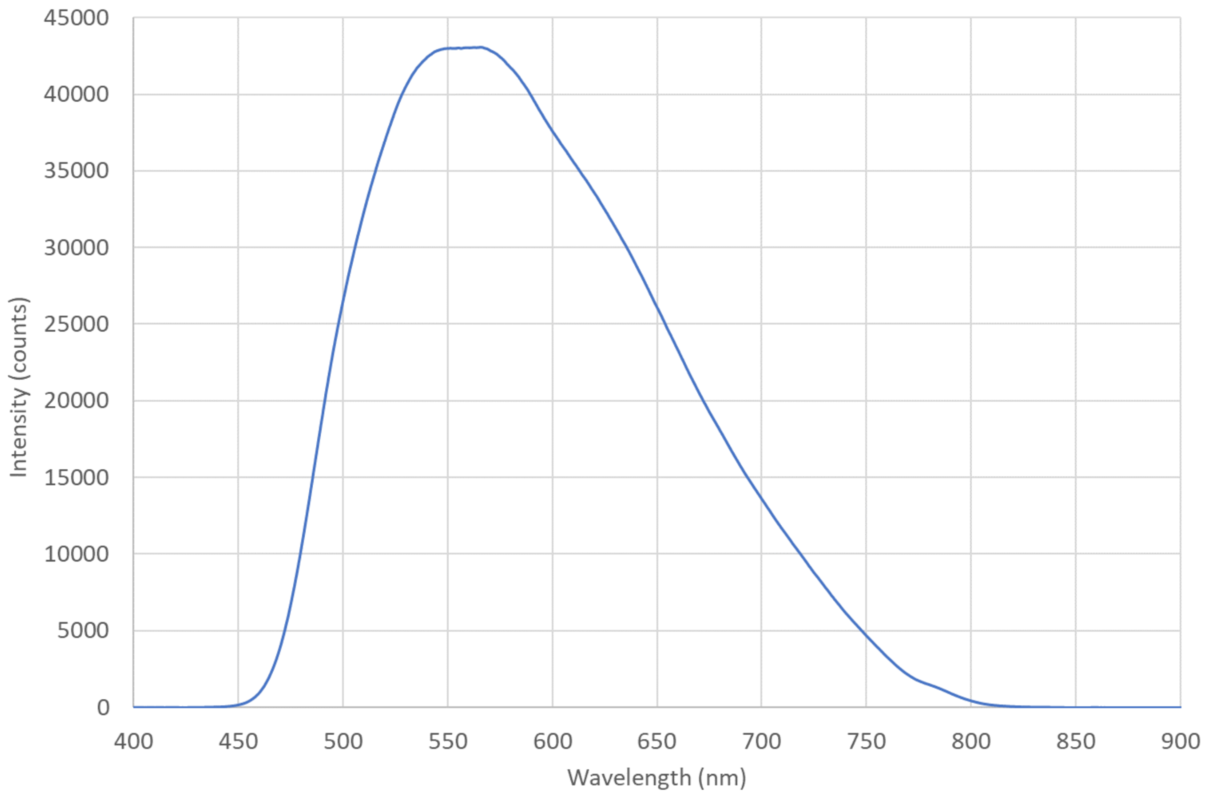

Fig. S9. Measured spectrum of bare gold film under bright field illumination.

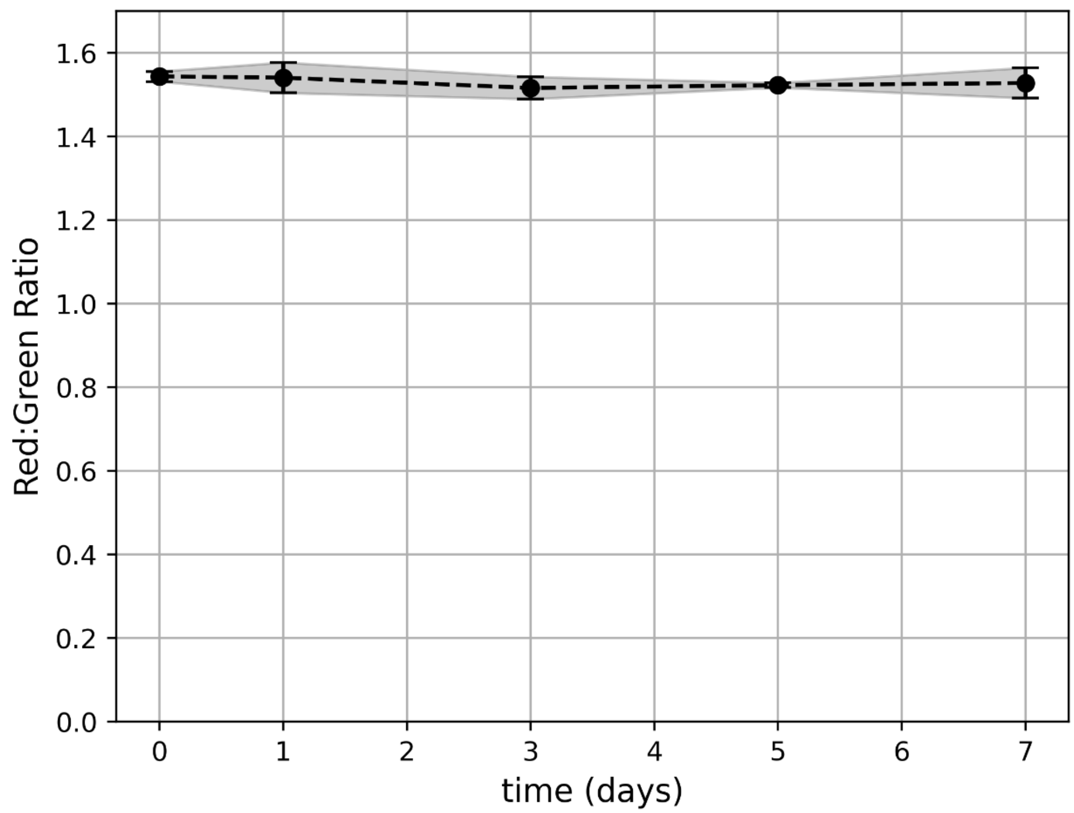

Fig. S10. Stability. Sensors were prepared on day 0 and stored in ambient air and room temperature. Upon the indicated time points, three sensors were challenged with endotoxin 500 EU/mL and the RG ratio was recorded.
